# Interesting Scenarios during Radiofrequency Ablation of Varicose Veins at University Hospital of Nepal

**DOI:** 10.1155/2020/2035494

**Published:** 2020-11-12

**Authors:** S. Vaidya, R. M. Karmacharya, A. K. Singh, P. Thapa

**Affiliations:** Department of Surgery (Cardiothoracic and Vascular Unit), Dhulikhel Hospital, Kathmandu University, Dhulikhel, State 3, Nepal

## Abstract

A varicose vein is a common venous condition which affects the great saphenous vein and small saphenous vein causing symptoms of pain, edema, itchiness, pigmentation, and ulceration. There are various modalities of the treatment of varicose veins; however, radiofrequency ablation is among the tested and proven treatments for varicose veins. With every case, there can be some unexpected or interesting scenarios which can pose both technical and surgical difficulties. The main objective of this paper is to introduce these scenarios which can occur despite following the standard protocol and methods both preoperatively and intraoperatively. In these scenarios, the surgeon quickly need to decide how to deal with the aberrations. Based on extensive literature and consensus of a team of three vascular surgeons, lists of interesting scenarios were prepared along with their definition. Any occurrences of such scenarios were noted in the operation theatre note. Here, we describe 39 (6.38%) interesting cases among 611 cases of radiofrequency ablation that was performed in Dhulikhel Hospital, Kathmandu University Hospital, from January 2014 until December 2019. Despite following the proper protocol, we can face many unexpected challenges preoperatively, peroperatively, and postoperatively. From this article, we concluded that vigilance of all the factors and proper Doppler ultrasonography can help in identifying most of these scenarios and aid in making proper surgical planning.

## 1. Introduction

A varicose vein is a common venous condition which affects the great saphenous vein and small saphenous vein which can cause pain, edema, pigmentation, itchiness, and ulceration depending upon the severity of the condition [1]. The great saphenous vein is the longest vein originating from the dorsum of the foot at the medial malleolus to the inguinal ligament [2]. Varicose veins have various modalities of surgical treatment ranging from open surgical treatment to endovenous treatment modalities [1]. Radiofrequency ablation is an endovascular treatment modality which is a proven and tested method [3]. Among many studies, Rasmussen et al. have done a randomized clinical trial between endovenous laser ablation and radiofrequency ablation which suggested that radiofrequency ablation has less periprocedural pain, analgesic requirement, and bruising. They also suggested that radiofrequency ablation has less technical failure and early recovery with less postoperative pain [4]. There are certain sets of standard steps followed for RFA of varicose veins with some minor modifications in each center [5]. Despite following all the standard protocols preoperatively and during radiofrequency ablation of varicose vein surgery, we can face a variety of scenarios and challenges. In these scenarios, the surgeon quickly needs to decide how to deal with the aberrations. There are only few reports of such scenarios in the literature causing the decision-making process even more difficult. We report such interesting scenarios during our experience of RFA for varicose veins from August 2014 till December 2019 with experience of a total of 611 cases.

## 2. Methodology

All the cases subjected for radiofrequency ablation (RFA) of varicose veins at Dhulikhel Hospital, Kathmandu University Hospital, from January 2014 till December 2019 were included in the study. All cases underwent preoperative and intraoperative ultrasonography using Siemens (Siemens Medical Solutions USA Inc.) P300 ultrasound with a linear probe (4-13 MHz). If any interesting scenarios were detected, then findings were included in the study. Similarly, during the procedure, despite following the standard procedural protocol, if any significant scenarios occurred which are not commonly expected, these were mentioned in the operation note. These occurrences were discussed independently by three vascular surgeons and, following the acceptance of all, are included for the analysis. Scenarios resulting due to surgical error or technical error were not included in the analysis.

Based on extensive literature and consensus of the team, lists of interesting scenarios were prepared as shown in Table 1 along with their definition.

RFA was done using a venous closure fast RF generator with an RFA probe of 60 cm or 100 cm in length. 2 cm distance was maintained between the RFA catheter tip and SFJ, between which 10 cc of normal saline was injected between the GSV and skin with the help of ultrasonography. And about 10 cc/cm normal saline was injected between the GSV and skin. Siemens (Siemens Medical Solutions USA Inc.) P300 ultrasound with a linear probe (4-13 MHz) was used for ultrasonography with appropriate gain and zoom.

Also, in patients in whom surgery was performed for bilateral lower limbs in the same setting, they were taken as separate cases, and occurrence of interesting scenarios specific to each limb is analysed separately for ease in interpretation.

A database was maintained in Microsoft Access 2013 (Microsoft Corporation) which was later copied in SPSS 13.0 (IBM), and necessary analysis was done. Frequency analysis was done and was expressed in number and percentage.

## 3. Results

There were 611 cases subjected to RFA for varicose veins during the time frame. We noticed 39 numbers (6.38%) of interesting scenarios (Table 2). None of the patients had two or more interesting scenarios at the same procedure. Table 2 shows the interesting scenarios we encountered.

### 3.1. Double Great Saphenous Vein

**Figure 1:**
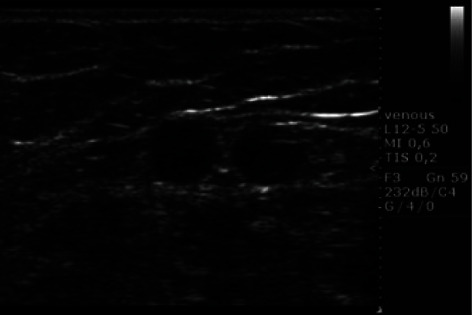
Double great saphenous vein.

**Figure 2:**
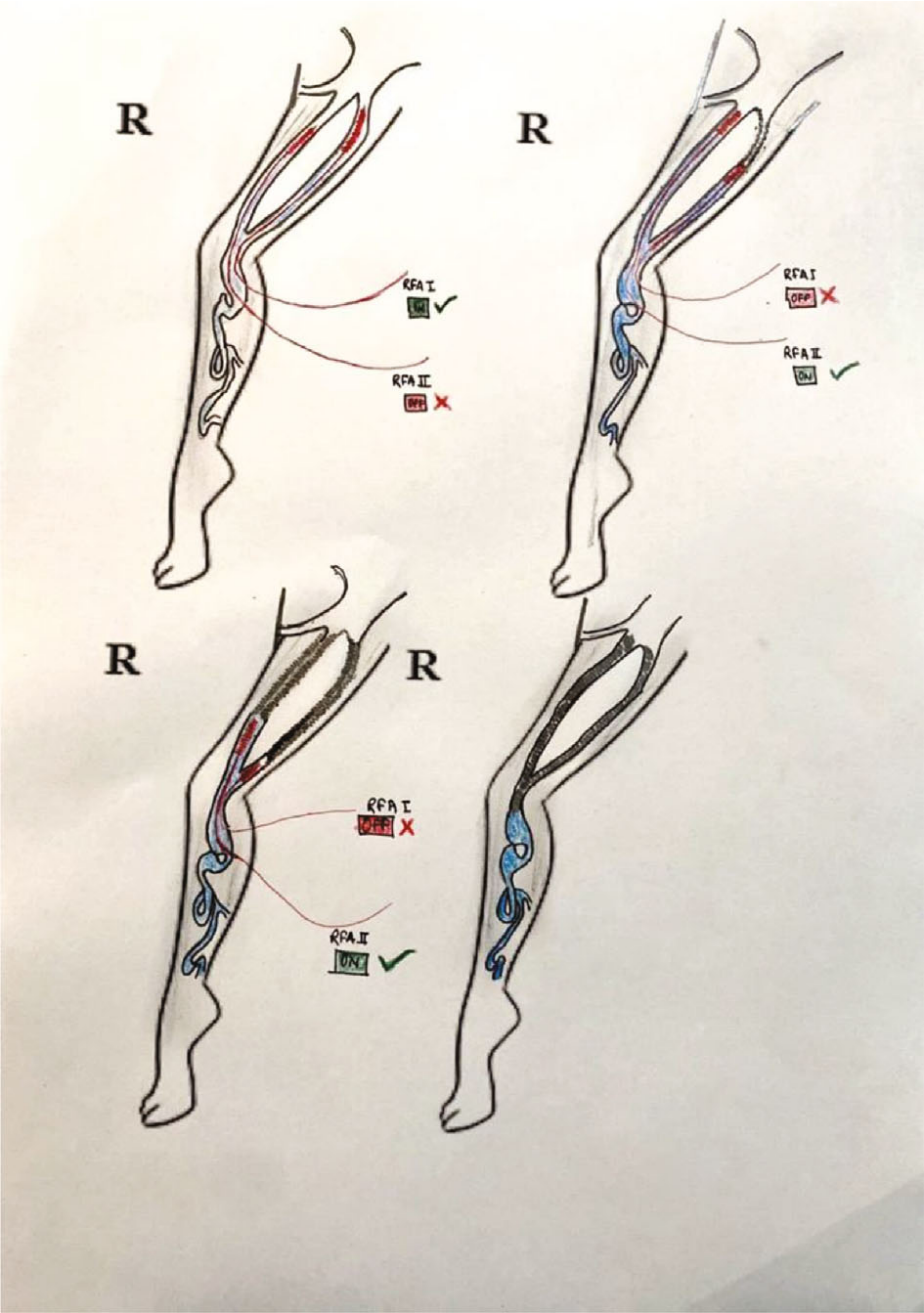
Double great saphenous vein with an alternating RFA technique.

**Figure 3:**
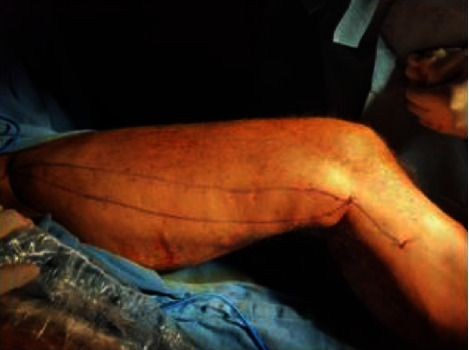
Marking of double GSV.

**Figure 4:**
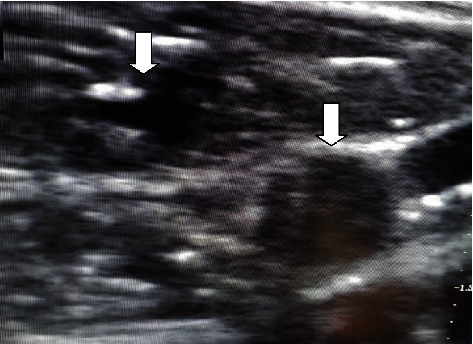
Thrombosed GSV and another GSV with a probe in situ.

Among 39 interesting cases, 4 (10.25%) of the cases belonged to double great saphenous vein (Figure 1). Between four cases, double GSV is missed in one case (25%) who presented to us with recurrence after which we performed RFA and the remaining three cases (75%) were detected early for which we performed RFA using an alternative technique. In an alternating RFA technique, cannulation is done in a usual technique in both the GSVs. This is followed by insertion of an RFA catheter in both the GSVs, and about 2 cm distance is maintained from the saphenofemoral junction. After that, normal saline is injected between the GSV and skin (for both the GSVs). While RFA is being done in the first GSV, pulling of an RFA probe (by about 10 cm) is done in the second GSV. After first-segment RFA in the first GSV is done, it is pulled by a segment (6.5 cm), and then, RFA in the second GSV is advanced and ablation is done. This cycle is repeated in both GSVs so that all desired segment RFA is done. As RFA is done in two GSVs alternately, this is termed as an “alternating RFA technique” (Figures 2–4). Thus, there is minimal chance of damage to the second RFA catheter during the operation of the first RFA catheter by doing alternating RFA over the two catheters. This however requires careful planning of sequential withdrawal of the catheters during the procedure.

### 3.2. “Shy GSV Phenomenon”

This term has not been coined in many literatures that we have searched. However, in many GSVs, we have found the incidence of spasm of GSV. We have found 15 (38.546%) cases of such GSV; for these cases, we have undergone an open cannulation technique and an RFA probe was inserted with this technique.

### 3.3. Recurrence due to a Lymph Node Vessel

**Figure 5:**
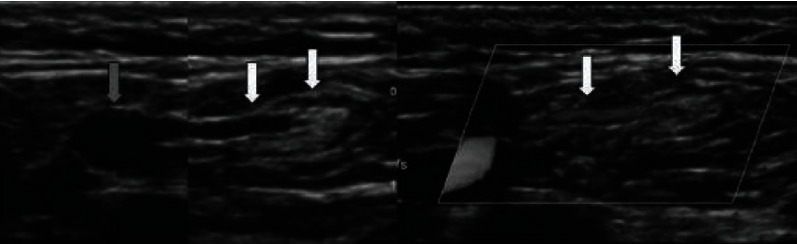
Lymph node vessel draining a lymph node.

**Figure 6:**
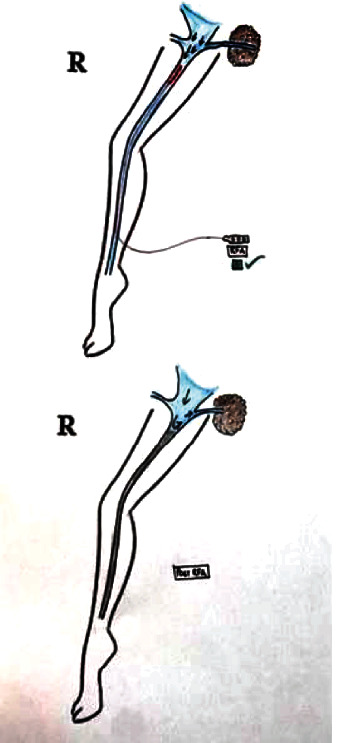
Schematic diagram showing a vessel draining a lymph node and RFA of GSV sparing a lymph node vessel.

**Figure 7:**
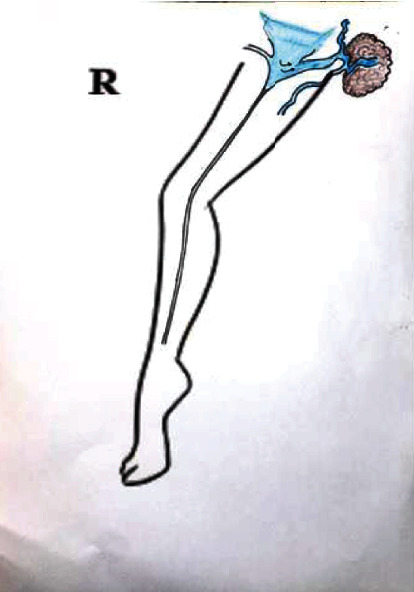
Schematic diagram showing recurrence of varicose veins around the groin region due to a lymph node vessel.

Another interesting phenomenon that we have found during our experience in the treatment of varicose veins was the recurrence of varicose veins after performing RFA which was seen specially on the groin region (Figures 5 and 6). One case was found among 39 cases which is 2.56%. For this case, we performed ligation of the lymph node vessel using an open technique and with the help of Doppler ultrasonography (Figure 7).

### 3.4. GSV Too Tortuous/Angulated or Thrombosed

**Figure 8:**
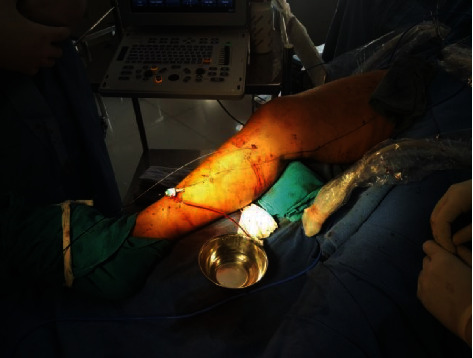
Double-prepuncture technique for RFA of the great saphenous vein with the tortuous segment in between two RFA catheters.

**Figure 9:**
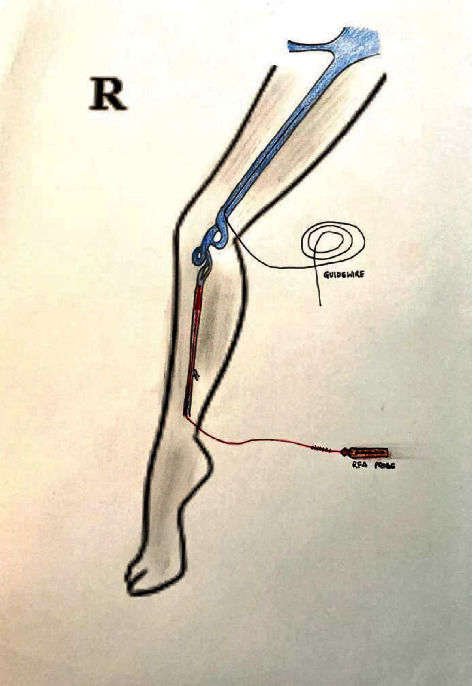
Tortuous GSV needing a double-prepuncture technique.

Among 39 cases, 12 (30.76%) cases presented with great saphenous vein tortuous/angulated or thrombosed. In these cases where GSV proximal to cannulation is too tortuous or angulated such that RFA cannot be inserted more proximally, we followed straightening of the knee joint, laterally pulling of thigh muscles, and a gentle pull-push RFA catheter [5]. In cases where advancement is difficult due to a catheter passing to the tributaries, we did an ultrasound-guided external compression of the tributaries so that the catheter passes to the proximal GSV. However, if these techniques fail, a double-prepuncture technique can be used [10]. After cannulating distal GSV, another cannulation of proximal GSV was done as proximal GSV might go into spasm if RFA is done right away and it might be difficult to puncture or insert a guide wire later (Figure 8). There was no recurrence, and the patient is also clinically well in all the 12 cases of RFA done by a double-prepuncture technique (Figure 9).

### 3.5. GSV and Deep System Too Close (<1 cm)

**Figure 10:**
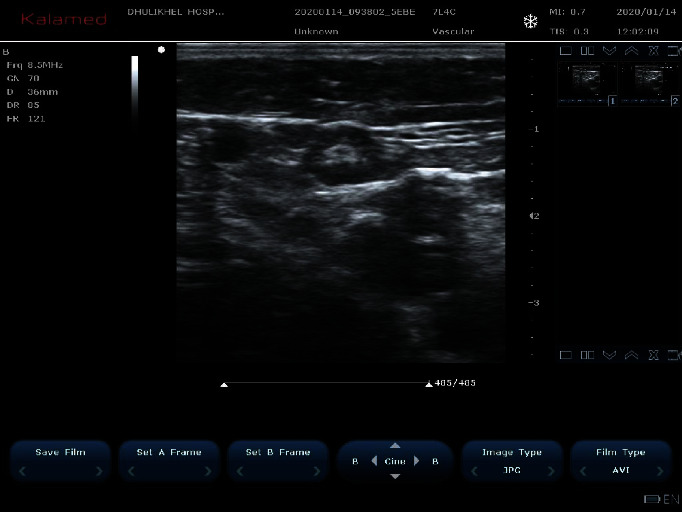
Triangle made by the GSV, lymph node, and deep veins.

**Figure 11:**
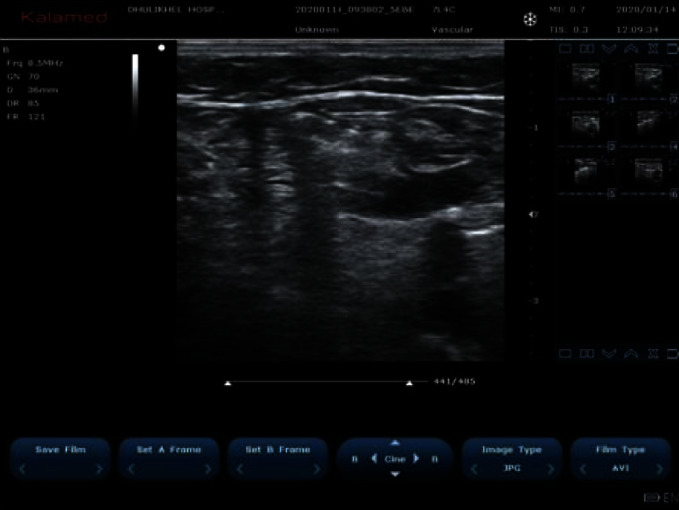
Increase in distance of instillation of normal saline.

A total of 7 (17.94%) cases were identified among 39 cases of the interesting scenario. These were diagnosed during the period of the RFA procedure and after the insertion of the RFA probe. We regularly measure the distance of GSV and deep vein during the RFA procedure, and these are done usually to prevent DVT in these patients. For the remedy of this problem, we install 10 cc of normal saline between the GSV and deep vein making a triangle with lymph node (Figure 10). This will increase the space between the GSV and deep vein and also prevent the DVT (Figure 11).

## 4. Discussion

### 4.1. Double Great Saphenous Vein

The great saphenous vein is a large, subcutaneous, and superficial vein of the lower limb [11]. However, in some incidences, there are two parallel great saphenous veins which lie in the same plane, parallel to the skin, and run along the aponeurotic deep fascia. The incidence of having a double great saphenous vein is 1.3% [12]. Although the short segment of GSV can commonly be doubled in the calf or thigh, having whole double GSV is not that common [13]. If two GSVs have a similar diameter draining a common cutaneous territory and opening directly into saphenofemoral junction, it is commonly termed as a double great saphenous vein [6], or when two veins course within the saphenous compartment and are connected by the saphenous ligament, it is termed as a double great saphenous vein [7]. These two GSVs will also have the same diameter draining a common cutaneous territory which is commonly termed as a double great saphenous vein. Sometimes, anterior and posterior accessory veins and double great saphenous veins are commonly confused. Anterior and posterior saphenous veins and double great saphenous veins are usually differentiated from their diameter, which is smaller and does not drain the same cutaneous territory as the great saphenous vein [6].

Here, we report four cases of a double great saphenous vein among 611 cases between the periods of 5 years. A double great saphenous vein was missed in one case who presented to us with recurrent dilatation of the vein after radiofrequency ablation on the same site. Kurt et al. in 2014 also reported a similar type of case which presented to them with recurrence after performing RFA. Similarly, we also performed radiofrequency ablation of the double great saphenous vein for the recurrence of varicose veins [14]. Similarly, three cases were identified preoperatively and alternative radiofrequency ablation was done using two radiofrequency ablation probes. Two probes were entered simultaneously in the same setting, and the first one great saphenous vein was ablated and consecutively another was ablated. In all three cases, there was no recurrence in follow-up.

Among many literatures, we reviewed about the need of treatment of both the GSVs or how often the duplicate GSV can become incompetent after treatment of incompetent GSV. But among the many causes of recurrence of varicose veins, the double great saphenous vein of the duplicate great saphenous vein is regarded as one. And we also had an experience of recurrence with the double great saphenous vein, so we planned for the treatment of the entire double great saphenous vein with the above-mentioned alternating technique. However, precise preoperative planning with duplex ultrasonography mapping can contribute to the better treatment of the patient and less chance of recurrence.

### 4.2. Shy GSV Phenomenon

It is well known to all that veins do not usually go to spasm; however, we have found in our interesting scenarios that the great saphenous vein tends to go into spasm. Many literatures were reviewed regarding any publications or case report for spasm of the vein; however, not many were found.

Few cases of vasospasm causing difficulty in cannulation in some cases has been documented [5]. Similarly, cases of the axillary vein going into spasm during contrast-guided puncture for pacemaker lead implantation have been reported [15]. But no any case report or any literature has been published for the great saphenous vein going into spasm during cannulation.

A strategy in such case is more proximal cannulation [5]. However, in some cases, we have observed repeated vasospasm causing cannulation impossible. A typical sequence in such cases is that following cannulation, during guide wire insertion, there are spasm of the vein and displacement of the guide wire while inserting it. The guide wire thus is in the juxta vein position. We propose the term “shy” GSV phenomenon for such finding. During this, we then try with open cannulation. If this also fails, open ligation of the saphenofemoral junction is needed. This phenomenon happened in 15 cases, all of which we did by open cannulation. An interesting finding we noted is that during open cannulation, although the size of GSV is small, there was no difficulty inserting the RFA probe via the segment which is in spasm.

### 4.3. Recurrence due to a “Lymph Node Vessel”

**Figure 12:**
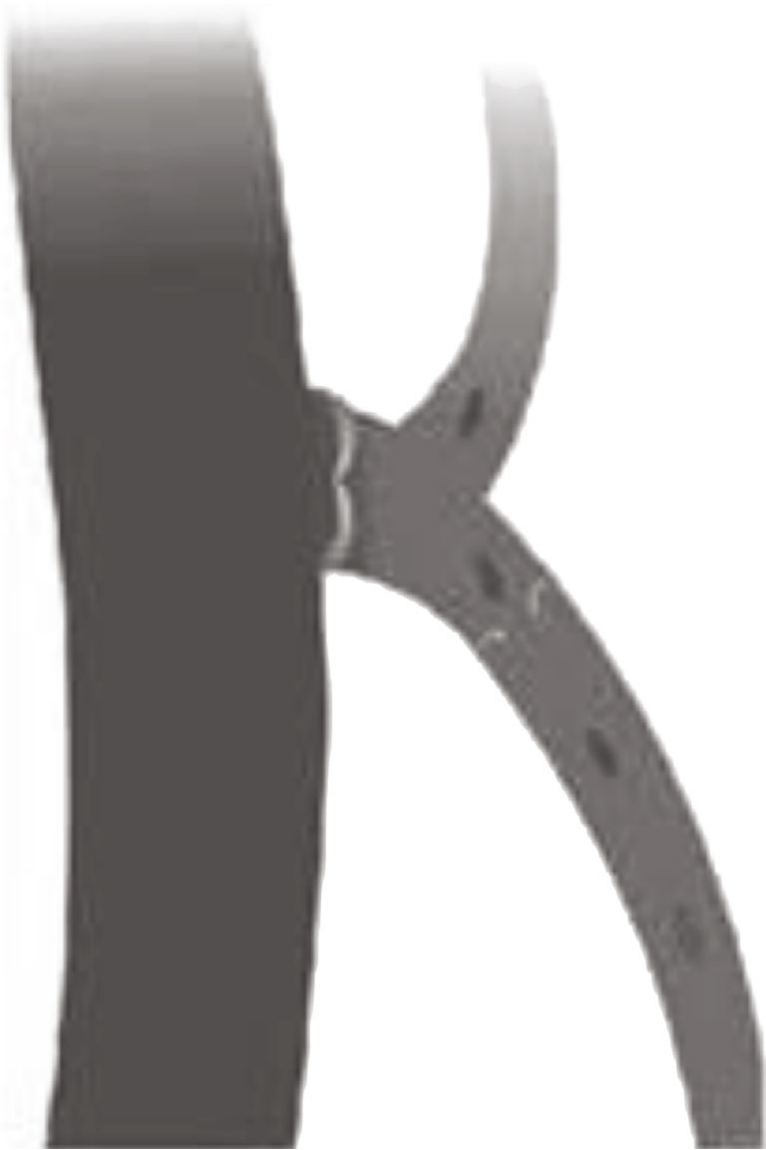
Incompetent GSV due to arch tributaries.

Lymph nodes are an integral part of the venous system of the body, especially in Scarpa's triangle. It communicates in many points in both superficial and deep venous systems of the body and in some cases may cause the incompetence of the superficial system of the body or may cause recurrence of varicose veins after radiofrequency ablation, stripping, or laser therapy. The saphenofemoral junction can be defined as the femoral venous segment limited by supra- and infrasaphenous valves, as well as by terminal and subterminal valves of the great saphenous vein arch [16]. Proximal tributaries that drain between the subterminal and terminal valves are the superficial circumflex iliac vein (lateral pathway), superficial epigastric vein (cranial pathway), and external pudendal vein (medial pathway). These veins can drain directly into the common femoral vein so they can be an important factor for the recurrence of varicose veins especially around the groin region after RFA, especially in situation where there are competent terminal valves and incompetent subterminal valves (Figure 12) [8].

Similarly, lymph nodes also have a tortuous venous system, which has a size between 1 and 3 mm in diameter, located between the anterior accessory saphenous vein and great saphenous vein. These veins are connected cranially, 10-15 cm proximal to the great saphenous vein. These are also difficult to find in duplex ultrasonography. Lymph nodes are also the part of Scarpa's triangle which connects the superficial venous system to the deep venous system [8].

A similar one case was presented to us with a recurrent varicose vein after the radiofrequency ablation procedure for the incompetent saphenofemoral junction. She presented to us with dilatation of veins in the great saphenous vein territory one month after radiofrequency ablation. She underwent sclerotherapy of the lymph node vessel under an ultrasound duplex scan, after which her symptoms subsided. A similar case was reported by Romualdo et al. in 2008 which also suggested a recurrence of a varicose vein after varicose vein stripping or endovenous ablation [17].

### 4.4. Tortuous or Angulated GSV

In some case where GSV proximal to cannulation is too tortuous or angulated such that RFA cannot be inserted more proximally, we follow straightening of the knee joint, lateral pulling of thigh muscles, and a gentle pull-push RFA catheter [5]. In cases where advancement is difficult due to a catheter passing to the tributaries, we do ultrasound-guided external compression of the tributaries so that the catheter passes to the proximal GSV. However, if these techniques fail, a double-prepuncture technique can be used [10]. After cannulating distal GSV, another cannulation of proximal GSV should be done as proximal GSV might go into spasm if RFA is done right away and it might be difficult to puncture or insert a guide wire later. There was no recurrence, and the patient is also clinically well in all the 10 cases of RFA done by a double-prepuncture technique. Joh et al. in 2014 also suggested a similar technique in the article published in 2014 with the title “Consensus for Treatment of Varicose Vein with Radiofrequency Ablation.”

### 4.5. GSV and Deep System Too Close (<1 cm)

In some cases, we found that GSV and deep systems are too close even below saphenofemoral junctions. If the two veins are too close (<1 cm), there are certain risks during RFA. A consensus document has advised at least 1 cm between the GSV and skin after perivenous fluid installation [5]. During the installation of normal saline between the GSV and skin, the GSV can be even closed to deep veins. If RFA is done in this manner, there is a chance of damage to the deep veins and arteries. In such a scenario, we propose two options. One way is to inject sufficient fluid (normal saline, 10 cc) between the GSV and deep veins first so that the distance is more than 1 cm followed by installation of fluid between the skin and GSV. After this, RFA can be safely done. This should be repeated for all the segments. Another option is to do conventional open surgery if there is considerable risk to the deep vessels. In seven cases, 4 were done in first technique while in 3 cases, conventional open surgery was done.

## 5. Conclusion

Although radiofrequency ablation is a simple procedure and counted as a day care basis of surgery, best results can be achieved using proper methods and algorithms. Despite following the proper protocol, we can face many unexpected challenges preoperatively, peroperatively, and postoperatively. Vigilance of all the factors and proper Doppler ultrasonography by the skilled surgeon are the key for the better and successful outcome of surgery.

## Figures and Tables

**Table 1:** Possible interesting scenarios during RFA of varicose veins.

S.N.	Interesting scenarios	Definition
1	Double GSV	If two GSVs have a similar diameter draining a common cutaneous territory and opening directly into the saphenofemoral junction, it is commonly termed as a double great saphenous vein [6]. In double GSV, two GSVs course within the saphenous compartment and are connected by the saphenous ligament [7] (Figure 1)
2	“Shy GSV phenomenon”	There is no such terminology we could find even after an extensive literature search. However, in some cases, we have found that great saphenous veins tend to go into spasm after puncturing with a cannulation needle during RFA. Even while trying proximal cannulation, the spasm reoccurs in some cases making percutaneous cannulation impossible. We are using the term “shy GSV phenomenon” for this phenomenon
3	Recurrence due to a lymph node vessel	This is the vessel located in the lymph nodes in Scarpa's triangle between the anterior accessory saphenous vein and great saphenous vein, which has a size of 1-3 mm in diameter and is tortuous in nature [8]. These veins directly drain into the deep venous system and can be a reason for recurrence of varicose veins in some cases [8, 9]
4	Tortuous or angulated great saphenous vein leading to a double-puncture technique	Tortuosity of veins below the knee and above the knee and angulation of veins at the level of the distal thigh
5	GSV and deep system too close	We like to highlight this scenario and have termed GSV close to the deep system if distance is less than 1 cm. The significance of this is that during RFA, it can cause injury to deep veins and cause DVT.

**Table 2:** Interesting scenarios during RFA of varicose veins.

S.N.	Interesting scenarios	Number of cases	Remarks
1	Double GSV	4	It was missed in one case which resulted in early recurrence. In the remaining three cases, RFA was done using an “alternate RFA technique” (Figure 2)
2	“Shy GSV phenomenon”	15	For these cases, we tried for two settings of puncture with a cannula. If cannulation failed due to spasm, then we opted to use an open method for cannulation. RFA is done in the proximal segment, and ligation is done in the distal part
3	Recurrence due to a lymph node vessel	1	Ligation of the lymph node vessel was done after identification of the vessel with the help of ultrasonography
4	GSV too tortuous/angulated or thrombosed so that single-catheter RFA could not be done	12	A double-prepuncture technique (Figure 12) was done in 10 cases. In the first two cases as this technique was not used, it was difficult to do proximal cannulation following RFA of the distal segment
5	GSV and deep system too close (<1 cm)	7	A total of 7 cases were identified and were treated with infusion of 10 cc normal saline to increase the space between the GSV and deep system which increases the distance and also prevents the DVT

## Data Availability

As per the hospital rules and regulations, the data is confidential.
